# Effect of digital mindfulness on perceived stress and anxious emotion among college students

**DOI:** 10.3389/fpsyt.2025.1644370

**Published:** 2025-08-01

**Authors:** Weirui Xiong, Xia Yu, Lu Yu, Fan Yang

**Affiliations:** School of Educational Science, Chongqing Normal University, Chongqing, China

**Keywords:** digital mindfulness, perceived stress, anxiety, primary care, mental health

## Abstract

**Background:**

Perceived stress and anxiety are common psychological issues among college students. Traditional mindfulness interventions effectively ameliorate these psychological issues, while digital mindfulness interventions—an emerging approach—are gaining increasing attention owing to their convenience and accessibility.

**Purpose:**

This study aimed to assess the effectiveness of digital mindfulness in reducing perceived stress and anxiety among college students and to explore its potential effects on mindfulness.

**Methods:**

The study employed a randomized controlled trial design and recruited 310 university students aged 18–22 years, randomly assigned to the intervention (*N*=156) or control (*N*=154) groups. The intervention group completed 4 weeks of mindfulness practice, engaging in 15–20 min guided sessions via social media 2–3 times per week. The control group did not receive any mindfulness training. The Chinese Perceived Stress Scale, Generalized Anxiety Disorder-7 scale, and Five Facet Mindfulness Questionnaire were used to assess participants’ stress perceptions, anxiety, and mindfulness levels at baseline and the end of the intervention.

**Results:**

At the end of the intervention, the intervention group reported significantly lower levels of perceived stress and anxiety and considerably higher levels of mindfulness. In contrast, the control group showed no significant changes in perceived stress, anxiety, or mindfulness levels.

**Conclusions:**

The 4-week digital mindfulness intervention effectively reduced perceived stress and anxiety levels and increased mindfulness among college students.

## 1 Introduction

### 1.1 Background of the study

The mental health of college students has always been a key topic of concern in schools and society. With the intensification of social competition, the psychological pressure on college student groups is increasing, and these pressures may lead to psychological distress and mental health problems. Perceived stress refers to an individual’s subjective assessment of how uncontrollable or difficult life events appear to be (1). According to a survey in China, approximately 67.5% of university students experience medium to high levels of perceived stress (2). These stressors cover many factors, such as academic pressure, interpersonal relationships, and future planning. At the physiological level, perceived stress negatively impacts sleep quality—higher levels of perceived stress are associated with poorer sleep quality (3). Psychologically, perceived stress is strongly linked to depression and anxiety. Research indicates that perceived stress is one of the primary triggers of anxiety (4). The higher the perceived stress levels of college students, the more severe their anxiety (5). Anxiety is an emotional state characterized by worry, unease and tension, usually accompanied by physical and cognitive changes (6). Although college students have basically completed their physical development, their psychological development is still relatively lagging behind, and the detection rate of anxiety is significantly higher than that of other groups. Worldwide, it is estimated that 12-50% of college students have anxiety (7). Therefore, identifying scientific and effective interventions to help college students manage stress better and reduce their anxiety levels is of great practical significance.

Mindfulness is a state of consciousness that is purposefully aware of the present moment without evaluation or judgment (8). Mindfulness has received widespread attention as a psychological intervention for relieving stress and enhancing mental health. In their studies addressing the effects of mindfulness training on improving individuals’ negative emotions, Xu et al. (9), Yu et al. (10), and Liu et al. (11) showed that mindfulness training can effectively reduce participants’ negative emotions. Liu et al. (12) investigated the effects of mindfulness meditation on mindfulness, spirituality, and subjective well-being of flight attendants. The results showed that mindfulness meditation positively affected participants’ subjective well-being and spirituality. Wu et al. (13) intervened with 95 college students in a depressive state using mindfulness training methods of different durations and contents. The results showed that all three methods significantly reduced the participants’ depression levels. Therefore, mindfulness interventions have a remarkable effect on improving an individual’s mental health.

With advances in time and technology, digital mental health services have gradually entered the public eye. Compared to traditional face-to-face formats, smartphone apps and web-based platforms have greater advantages in enhancing the accessibility, standardization, degree of personalization, and effectiveness of mindfulness training (14). Moreover, digital-based mindfulness practices have been proven to be effective. For example, research by Barcaccia et al. (15) indicates that short-term online mindfulness interventions can effectively alleviate depression, rumination, and trait anxiety among college students. Fazia et al. (16) found that online mindfulness intervention measures effectively reduced stress perception among medical students and improved their mental health and psychological resilience, among others. A meta-analysis of digital online mindfulness interventions also indicated that online mindfulness interventions significantly impacted stress and that the effect size was significantly moderated by the number of interventions (17). Chen et al. (18), Liu et al. (19) and others have conducted similar studies, arriving at the same conclusion. Chiou et al. (20) and Liu et al. (21) also developed an application based on the Integrated Service Digital Terminal and mindfulness with the theme of compassionate meditation to improve depression and enhance spirituality, which has been proven effective. Other studies have found that mindfulness meditation guided by short-video apps can effectively enhance police officers’ conflict resolution and communication skills, and improve their emotional disorders and post-traumatic stress disorder (22–24).

Digital mindfulness, as an emerging psychological intervention approach, has received extensive attention and application in recent years among clinical patient groups, such as those with severe psychological or physical disorders. It combines modern digital technology with the concept of mindfulness, providing convenient and accessible mindfulness exercises through digital platforms, thus offering a new avenue for psychological intervention. Based on this, this study aims to explore its potential and practical effects in alleviating stress perception and anxiety among college students, with the expectation of providing more contemporary and innovative solutions for mental health intervention in universities.

Hypothesis

Based on the above theories and research, this study proposes the following hypothesis:

A 4-week digital mindfulness intervention can reduce college students’ perception of stress and anxiety, and improve their mindfulness levels.

## 2 Methodology

### 2.1 Participants

This study adopted the method of convenient sampling and selected college students from two universities in Chongqing, China as the research subjects. Participants were enrolled voluntarily through campus recruitment advertisements, and at the end of the study, they were provided with activity credits as incentives for completing the activities. The inclusion criteria were as follows: (1) voluntary participation in this study, (2) no systematic training related to mindfulness, (3) no current use of anxiolytic medication or undergoing other forms of psychological intervention, and (4) the ability to participate in the entirety of this activity. A total of 328 eligible students were recruited, all of whom completed the pre-test questionnaire and were randomly assigned to the control and experimental groups, with 164 students in each group. A total of 18 students dropped out without taking the post-test, and the final sample consisted of 310 participants, 156 in the intervention group and 154 in the control group, aged 18–22 years (*M*=19.26, *SD*=0.79). All the participants provided informed consent.

### 2.2 Procedure

This study used a randomized controlled trial design in which all eligible participants were randomly assigned to the intervention and control groups and received a baseline questionnaire. The intervention group received digital mindfulness training for 4 weeks, 2 to 3 times a week, for 15–20 min each session. The number and duration of practice each week were selected and adjusted by the participants according to their own conditions. The control group received no specific psychological intervention during the study period but after the study, the subjects will be provided with the same digital mindfulness intervention materials as the intervention group and given guidance during the practice process. The training was posted online on social media. Based on the core theoretical framework of Kabat-Zinn’s Mindfulness-Based Stress Reduction and considering the college students’ cognitive characteristics and stress-related features, the researchers independently developed a series of mindfulness training scripts. They then recorded 10 instructional audio sessions, each lasting 15–20 min, using standardized video recording equipment. The content included mindful stretching, breathing, and body scanning, among others. The lead trainer released the audio sessions on Mondays, Wednesdays and Fridays via social media, and participants were instructed to complete the practice on the same day and record their activity using an online mini-program. The lead trainer regularly responded to participants’ questions and provided guidance online, monitored adherence to the exercises through online questionnaires and follow-ups via social media return visits, and collected feedback to allow timely adjustments to the training program. Before and after the intervention, the Chinese Perceived Stress Scale (CPSS), Generalized Anxiety Disorder (GAD-7) scale, and Five Factor Mindfulness Questionnaire (FFMQ) were administered to assess the perceived stress, anxiety, and mindfulness levels of participants in both groups. Data were collated and analyzed using SPSS Windows software version 22.0.

### 2.3 Measurement tools

#### 2.3.1 Chinese version of the perceived pressure scale

The Stress Perception Scale (SPS), developed by Cohen et al. (1), assesses individuals’ perceived stress in daily work and life. Yang and Huang (25) adapted the scale for Chinese populations. The scale comprises 14 entries and two dimensions: a sense of loss of control and sense of tension, with a five-point scale (0 = never, 4 = always) and a total score of 0-56, with higher scores indicating a higher degree of perceived stress. In this sample, the scale showed good internal consistency at both baseline and post-test, with Cronbach’s coefficients of 0.74 and 0.82, respectively.

#### 2.3.2 Generalized anxiety disorder scale

The GAD-7 scale was developed by Spitzer et al. (26) to assess an individual’s generalized anxiety symptoms over the past two weeks. The scale comprising seven items rated on a four-point scale (0 = not at all, 3 = almost every day), with a total score of 0-21, with higher scores indicating more severe anxiety symptoms. The scale has good reliability and is suitable for use in the Chinese population (27). In the present sample, the scale showed good internal consistency at both baseline and post-test, with Cronbach’s coefficients of 0.79 and 0.85, respectively.

#### 2.3.3 Five-factor mindfulness questionnaire

FFMQ was developed by Baer et al. (28) and translated and revised by Deng (29). The FFMQ comprises 39 items across five dimensions: observation, description, conscious action, non-judgment, and non-reaction. It is designed to assess individuals’ ability to pay attention to their inner feelings, focus on the present moment, and be non-judgmental about the present. The questionnaire was rated on a five-point scale (1 = complete non-conformity, 5 = complete conformity), with a total score of 39-195, with higher scores indicating higher levels of mindfulness. In the present sample, the scale showed good internal consistency at both baseline and post-test, with Cronbach’s coefficients of 0.70 and 0.77, respectively.

### 2.4 Statistical processing

In this study, data were entered using Excel and descriptive statistics, independent samples t-test, *χ²* test, and ANOVA were conducted using SPSS Windows software version 22.0.

## 3 Results

### 3.1 Test of homogeneity of differences between intervention and control groups

Before the intervention, independent samples t-test and *χ²* test were used to test the homogeneity of difference between the intervention and control groups, and the results are shown in **Table 1**. There were no significant differences between the intervention and control groups regarding age, sex, CPSS, GAD-7, or FFMQ (*p* >.05). Therefore, the two groups of participants were homogeneous and could be tested for the interventional effects.

**Table 1:** Test of homogeneous differences between intervention and control groups.

Variable	Variable category	Intervention group (*N*=156)		Control group (*N*=154)		Total (*N*=310)		Statistical analysis results
		*M*	*SD*	*M*	*SD*	*M*	*SD*	
Age		19.19	0.94	19.27	0.90	19.23	0.92	*t*= .84, *p*= .404
Sex	men	64	41.0%	65	42.2%	129	41.6%	*χ*²= .045, *p*= .833
	women	92	59.0%	89	57.8%	181	58.4%	
Outcome variable	CPSS	25.83	5.52	25.90	5.53			*t*= .10, *p*= .920
	GAD-7	5.56	2.60	5.55	2.86			*t*= -.04, *p*= .969
	FFMQ	119.48	9.37	120.55	9.99			*t*= .97, *p*= .334

CPSS refers to Chinese version of the Perceived Pressure Scale; CAD-7 refers to Generalized Anxiety Disorder Scale; FFMQ refers to Five-Factor Mindfulness Questionnaire.

### 3.2 Post-test difference tests for intervention and control groups

At the end of the intervention, the post-test data of the intervention and control groups were examined using an independent samples t-test, and the results are shown in **Table 2**. Significant differences existed between the intervention and control groups on CPSS, GAD-7, and FFMQ (*t*= 5.25, *p <*.001; *t*= 5.88, *p <*.001; *t*= -2.72, *p*= .007).

**Table 2:** Tests for differences between intervention and control groups at post-test.

Outcome variable	Intervention group (*N*=156)	Control group (*N*=154)	*t*	*p*
CPSS	21.94 ± 5.94	25.82 ± 7.03	5.25	<.000
GAD-7	3.51 ± 2.55	5.40 ± 3.09	5.88	<.000
FFMQ	123.36± 10.61	120.06± 10.69	-2.72	.007

CPSS refers to Chinese version of the Perceived Pressure Scale; CAD-7 refers to Generalized Anxiety Disorder Scale; FFMQ refers to Five-Factor Mindfulness Questionnaire.

### 3.3 Repeated measures ANOVA of perceived stress in intervention and control groups

**Figure 1:**
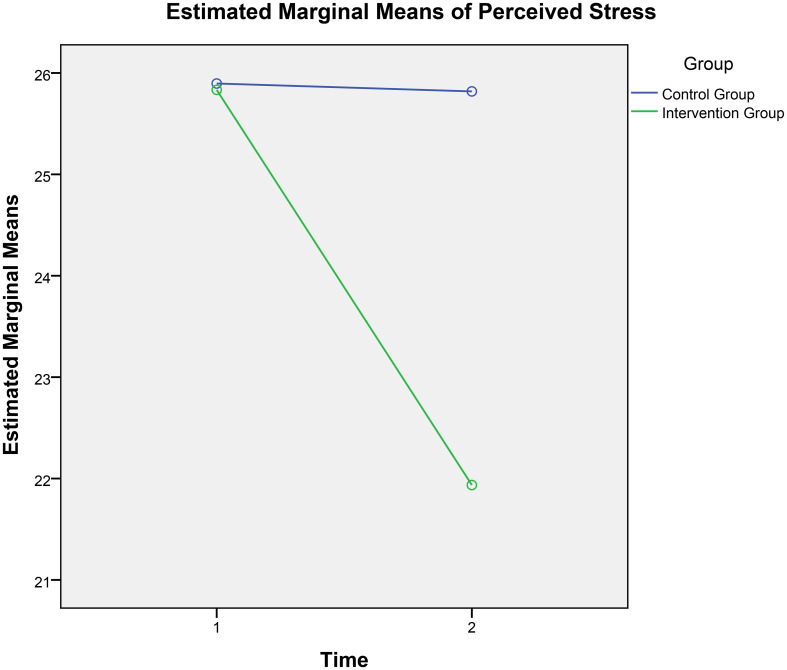
Time and group interaction perceived stress.

A 2 × 2 repeated-measures ANOVA was conducted with perceived stress as the dependent variable, time (pre-test and post-test) as the within-participants factor, and group (experimental and control) as the between-participants factor. As shown in **Table 3**, there was a significant main effect of measurement time (*F*[1, 308] = 38.90, *p <*.001, partial *η²* = .11), a significant main effect of group (*F*[1, 308] = 10.55, *p* = .001, partial *η*²= .03), and a significant interaction between group and time (*F*[1, 308] = 35.91, *p <*.001, partial *η*²= .10). The interaction is shown in **Figure 1**, indicating that the perceived stress score tends to change over time, and 4 weeks of digital mindfulness training can influence this trend.

**Table 3:** Repeated measures ANOVA for pressure perception in college students.

Effect	*F*	*p*	Partial *η²*
Group main effect	10.55	.001	.03
Time main effect	38.90	<.001	.11
Group × time main effect	35.91	<.001	.10

Further simple effects analyses showed no significant difference in pre-test scores between the intervention and control groups (*F*[1, 308] = .10, *p* = .92, partial *η²* = .000). However, there was a significant difference in perceived stress scores on the post-test (*F*[1, 308] = 27.58, *p <*.001, partial *η²* = .08), with the intervention group scoring significantly lower than the control group.

In the time dimension, no significant difference was found between the control group’s pre-test and post-test perceived stress scores (*F*[1, 308] = .03, *p* = .86, partial *η²* = .000). In contrast, the intervention group significantly reduced perceived stress from pre-test to post-test (*F*[1, 308] = 75.26, *p <*.001, partial *η²* = .20), with post-test scores substantially lower than pre-test scores (*p <*.001). These findings suggest that the 4-week digital mindfulness training program effectively reduced perceived stress among participants.

### 3.4 ANOVA for repeated measures of anxiety in the intervention and control groups

**Figure 2:**
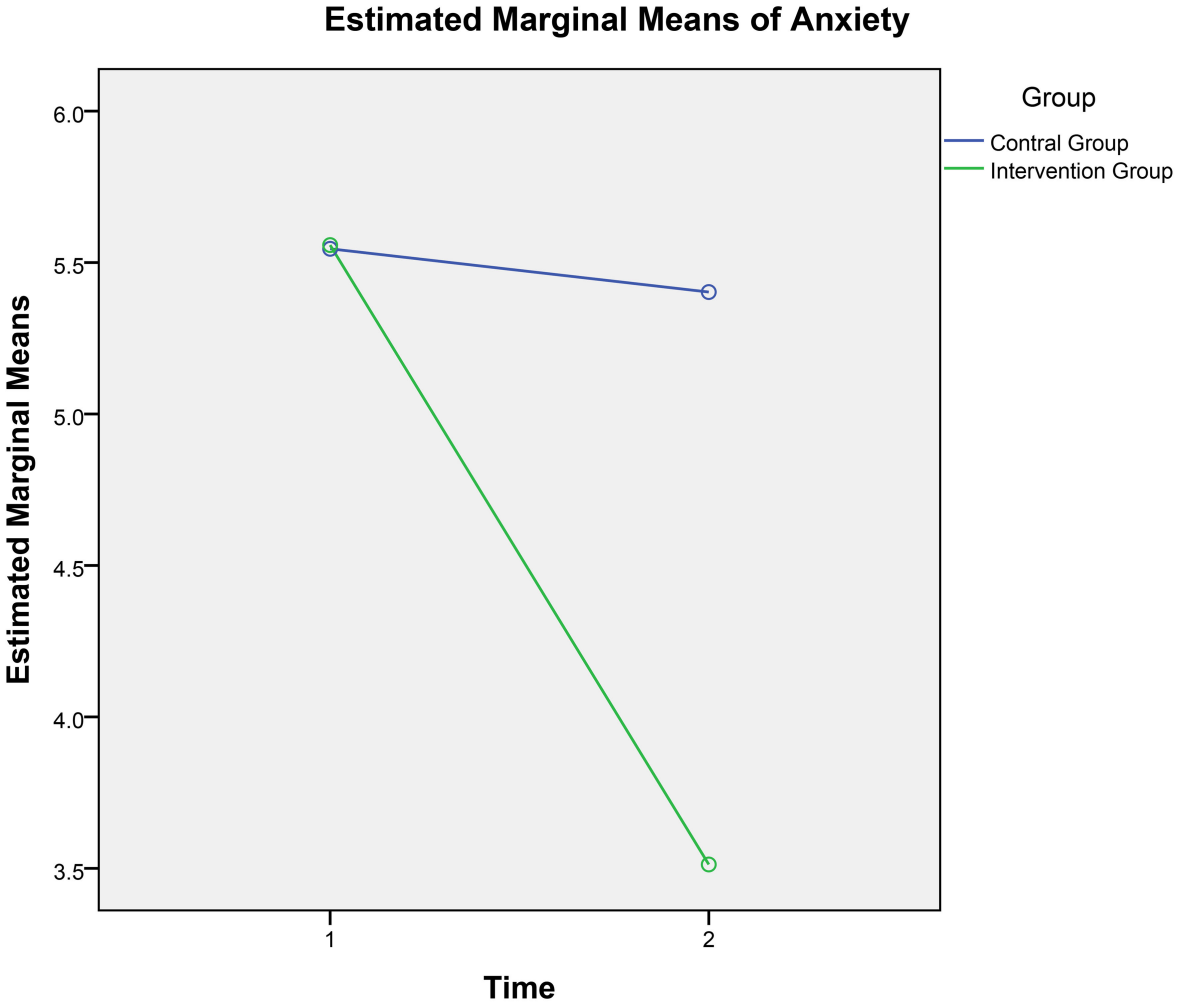
Time and group interaction for anxiety.

A 2 × 2 repeated-measures ANOVA was conducted with anxiety as the dependent variable, time (pre-test and post-test) as the within-participants factor, and group (experimental vs. control) as the between-participants factor. As shown in **Table 4**, there was a significant main effect of measurement time (*F*[1, 308] = 38.01, *p <*.001, partial *η²* = .11) and a significant main effect of group (*F*[1, 308] = 12.87, *p <*.001, partial *η*²= .04). Further, there was a significant interaction between group and time (*F*[1, 308] = 28.73, *p <*.001, partial *η*²= .09). The interaction is depicted in **Figure 2** and indicates that anxiety scores changed over time, with the 4-week digital mindfulness training influencing this trend.

**Table 4:** Repeated measures ANOVA for anxiety levels in college students.

Effect	*F*	*p*	Partial *η²*
Group main effect	12.87	<.001	.04
Time main effect	38.01	<.001	.11
Group × time main effect	28.73	<.001	.09

Further simple effects analyses revealed that, on the group dimension, there was no significant difference in anxiety scores between the intervention and control groups at the pre-test (*F*[1, 308] = .002, *p* = .97, partial *η²* = .000). However, at the post-test, a significant difference emerged (F[1, 308] = 34.51, p <.001, partial η² = .10), with the intervention group exhibiting significantly lower anxiety scores than the control group.

On the time dimension, there was no significant difference between the control group’s anxiety scores at pre-test and post-test (F[1, 308] = .32, p = .57, partial η² = .001). In contrast, the intervention group showed significant anxiety scores at pre-test and post-test (F[1, 308] = 66.85, p <.001, partial η² = .18), with post-test anxiety scores significantly lower than pre-test scores (p <.001). These results suggest that the 4-week digital mindfulness training program effectively reduced participants’ anxiety levels.

### 3.5 Repeated measures ANOVA for mindfulness in intervention and control groups

**Figure 3:**
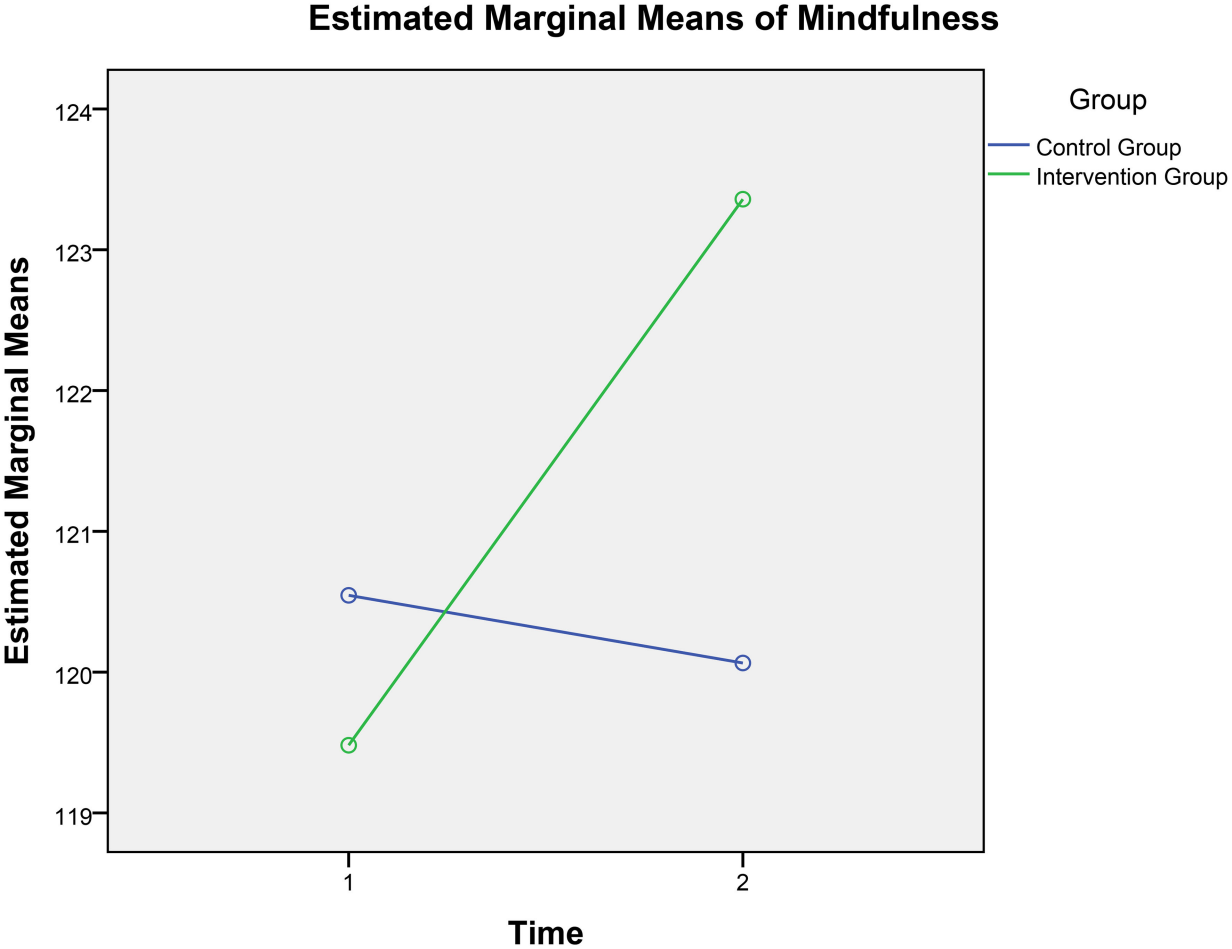
Time-group interaction for mindfulness.

A 2 × 2 repeated-measures ANOVA was conducted with mindfulness as the dependent variable, time (pre-test and post-test) as the within-participants factor, and group (experimental vs. control) as the between-participants factor. As shown in **Table 5**, the main effect of measurement time was significant (*F*[1, 308] = 10.22, *p*= .002, partial *η2* = .03). The main effect of group was not significant (*F*[1, 308] = 1.18, *p* = .297, partial *η2* = .04). However, the interaction between group and time was significant, (*F*[1, 308] = 16.81, *p <.*001, partial *η2* = .05). This interaction is illustrated in **Figure 3**, suggesting that the mindfulness scores tends to change over time, and that the 4-week digital mindfulness training program influenced this trend.

**Table 5:** Repeated measures ANOVA for mindfulness levels in college students.

Effect	*F*	*p*	Partial *η2*
Group main effect	1.18	.297	.04
Time main effect	10.22	.002	.03
Group × time main effect	16.81	<.001	.05

Further simple effects analyses revealed that, on the group dimension, there was no significant difference between the scores of the intervention and control groups on the pre-test (*F*[1, 308] = .94, *p* = .334, partial *η2* = .003). However, there was a significant difference between the intervention and control groups on the post-test for positive mindfulness scores (*F*[1, 308] = 7.42, *p* = .007, partial *η2* = .02). Specifically, the intervention group had significantly higher post-test scores than the control group.

On the time dimension, there was no significant difference between the control group’s pre-test and post-test mindfulness scores (*F*[1, 308] = 0.41, *p* = .524, partial *η2* = .001). In contrast, the intervention group showed a significant difference between pre-test and post-test of perceived stress (*F*[1, 308] = 26.79, *p <*.001, partial *η2* = .08). Specifically, the post-test mindfulness score was significantly higher than the pre-test (*p <*.001). These results indicate that the 4-week digital mindfulness training program effectively enhanced participants’ mindfulness levels.

## 4 Discussion

This study systematically explored the effects of a digital mindfulness intervention on perceived stress and anxiety among college students using a randomized controlled experiment. The pre-intervention independent samples t-test showed that the experimental and control groups were homogeneous regarding age, sex, and perceived stress levels, anxiety, and mindfulness (p >.05). The results of further repeated-measures ANOVA showed a significant group × time interaction (p <.001) for perceived stress, anxiety and mindfulness levels in the intervention and control groups. Specifically, the intervention group exhibited a significant decrease in perceived stress (and anxiety levels (p <.001), including a significant increase in mindfulness levels after the intervention. In contrast, the control group showed no significant changes across any of the three variables during the same time (p >.05).

On the perceived stress and anxiety dimensions, the experimental group’s post-test scores were significantly lower than those of the control group, with a significant group × time interaction effect observed, this result is consistent with the findings of Flett et al. (30), Huberty et al. (31), and Küchler et al. (32). The partial η² values were 0.10 and 0.09 respectively, reaching a moderate effect (33), suggesting that digital mindfulness training is effective in mitigating psychological distress among college students. Several studies have demonstrated that this effect occurs via multiple mechanisms. First, mindfulness training can help trainers change their perceptions of unpleasant emotions, feelings, or sensations, thereby reducing physiological indicators of anxiety (34). Second, mindfulness training improves trainers’ cognitive functioning and reduces wandering (35), which may allow them to focus more on the task they are currently performing in the present moment, thus indirectly reducing stress perceptions. Additionally, Mindfulness Stress-buffering Account holds that Learning to monitor experiences with an accepting attitude is an emotional regulation skill learned in mindfulness intervention, which can cultivate resilience and coping ability under pressure. Furthermore, these stress buffering effects, in turn, reduce the negative impact of stress on increasing the risk of stress-related diseases (36), which also explains why mindfulness intervention can lower the stress perception and anxiety levels of the subjects.

On the mindfulness dimension, the experimental group’s post-test scores were significantly higher than those of the control group, and the interaction effect between group and time was significant (partial η² = .05). This is consistent with previous findings (37, 38), suggesting that a digitally based mindfulness intervention can significantly improve students’ mindfulness. In particular, this is achieved by directing their attention to the present moment and developing their awareness of their internal experience and external environment. The results of repeated-measures ANOVA showed that the group main effect did not reach significance (p = .297) on the mindfulness dimension. However, the significant interaction (partial η² = .05) indicated that the improvement in the mindfulness level of the experimental group was closely related to the intervention. This apparent paradox may be explained by the fact that the group main effect reflects overall between-group differences across time points. Thus, opposing patterns in pre- and post-test scores may have offset each other, resulting in a non-significant overall difference. This finding also suggests that the improvement of mindfulness ability requires long-term practice to be transformed into stable trait changes (39).

The present study found that a 4-week digital mindfulness intervention can provide significant intervention effects on perceived stress and anxiety levels among college students. This provides important insights for psychological services in higher education. A cross-sectional survey of 500 adults in the United States suggested that many individuals prefer individual and online forms of mindfulness meditation interventions to group formats (40). This suggests that a digitally based approach to mindfulness interventions may be more in tune with university students’ usage preferences. Digital mindfulness interventions may be more flexible in both time and space and less costly than traditional intervention models that require face-to-face instruction (41). Specifically, digital mindfulness intervention is carried out through the Internet and mobile devices. Students can practice mindfulness at any time and any place. The time cost and economic cost of mindfulness practice have been significantly reduced, thereby enhancing the feasibility of the intervention and students’ compliance. However, traditional mindfulness methods usually require face-to-face practice and guidance at designated times and places, which is often difficult for college students with numerous courses to adhere to. In addition, the sources of stress and manifestations of anxiety for each college student may vary. The uniform practice plan of traditional mindfulness methods often fails to meet individual differences, and the effect is inevitably limited to a certain extent. However, the personalization of digital mindfulness practice can better meet the needs of different students. Therefore, incorporating digital mindfulness interventions into the mental health intervention systems of colleges and universities is of great practical significance for coping with the growing psychological stress and anxiety problems of the college student population. This innovative service model helps to expand the coverage of mental health services, enabling more students to benefit from them and promoting the development of campus mental health service systems towards diversification and personalization.

## 5 Study limitations

First, this study failed to conduct long-term follow-up measurements on the subjects. Due to a certain degree of subject attrition after the conclusion of the research, subsequent follow-up measurements could not be carried out smoothly. This limitation prevented us from conducting a comprehensive assessment of the sustained effects of the digital mindfulness intervention method. Second, the control group design of this study could not completely exclude the influence of the placebo effect or other nonspecific factors, such as life events and personal psychological traits of college students. Future research should consider setting up more positive control groups, such as psychological education intervention groups or non-mindfulness-based relaxation training groups, to evaluate the specific effects of digital mindfulness interventions more accurately. In addition, future research could incorporate multiple follow-up measurements after the intervention to examine the persistence and stability of the effects of digital mindfulness intervention.

## 6 Conclusion

The results of this study indicated that digital-based mindfulness practice can effectively reduce perceived stress and anxiety levels among college students and enhance their mindfulness levels. This result indicates that the digital mindfulness intervention has a significant effect on improving mental health, which provides strong support for the digital transformation of mental health services. Future research should further explore the specific mechanisms of digital mindfulness interventions and how to better integrate them into college students’ mental health education.

## Data Availability

The original contributions presented in the study are included in the article/supplementary material. Further inquiries can be directed to the corresponding authors.
